# Mayaro Virus Infection, Amazon Basin Region, Peru, 2010–2013

**DOI:** 10.3201/eid1911.130777

**Published:** 2013-11

**Authors:** Eric S. Halsey, Crystyan Siles, Carolina Guevara, Stalin Vilcarromero, Erik J. Jhonston, Cesar Ramal, Patricia V. Aguilar, Julia S. Ampuero

**Affiliations:** US Naval Medical Research Unit No. 6, Lima and Iquitos, Peru (E.S. Halsey, C. Siles, C. Guevara, S. Vilcarromero, J.S. Ampuero);; Universidad Nacional de la Amazonia Peruana, Loreto, Peru (E.J. Jhonston);; Hospital Regional, Loreto, Peru (C. Ramal);; University of Texas Medical Branch, Galveston, Texas, USA (P.V. Aguilar)

**Keywords:** alphavirus, arboviruses, Peru, arthralgia, chronic pain, diagnosis, viruses, vector-borne infections

## Abstract

During 2010–2013, we recruited 16 persons with confirmed Mayaro virus infection in the Peruvian Amazon to prospectively follow clinical symptoms and serologic response over a 12-month period. Mayaro virus infection caused long-term arthralgia in more than half, similar to reports of other arthritogenic alphaviruses.

Since the discovery of Mayaro virus (MAYV) in Trinidad in 1954, the etiologic agent of Mayaro fever has been identified in French Guiana, Suriname, Venezuela, Peru, Bolivia, and Brazil (*1*–*9*). The presumed primary vectors, *Haemagogus* mosquitoes, inhabit rural settings and tree canopies, a factor that may explain the relative paucity of cases and restricted endemicity. However, *Aedes aegypti* mosquitoes have been shown to be competent vectors of MAYV in the laboratory (*10*), suggesting that an urban-dwelling arthropod could be a vector of this virus over a wider scale. MAYV infection has been demonstrated in tourists returning from the Amazon region, highlighting not only the need to consider MAYV in febrile returned travelers, but also a possible role in global transmission (*11*).

Incapacitating chronic joint pain has been described with other arthritogenic alphaviruses (*12*), but little is known about the prognosis and serologic response over long periods after MAYV infection. Therefore, we conducted a prospective 1-year longitudinal study to determine the clinical manifestations and to describe the serologic response among humans with Mayaro fever in the Peruvian Amazon Basin.

## The Study

**Figure 1:**
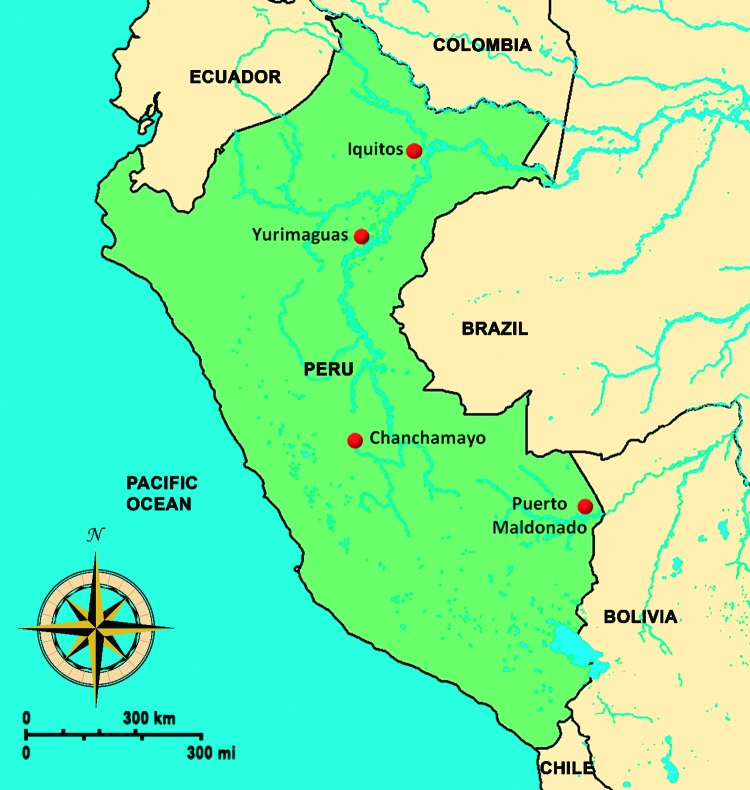
Map of Peru. Sites for study of Mayaro virus–infected patients are marked with a dot.

Persons identified for this cohort were recruited in a passive febrile surveillance study in 15 health centers in 4 Peruvian cities: Iquitos, Yurimaguas, Chanchamayo, and Puerto Maldonado (Figure 1). Persons meeting the following criteria were recruited: age >5 years, oral/tympanic temperature >38°C (or axillary >37.5°C), and no obvious focus of infection. Written consent was obtained from all adults and from a parent or guardian for participants <18 years of age; participants 8–17 years of age also provided written assent. The surveillance period of this study was December 6, 2010–April 30, 2012. Follow-up appointments continued for another year, through April 5, 2013. The institutional review boards of the US Naval Medical Research Unit No. 6 and the Peruvian Ministry of Health approved the protocol.

Compared with the day of the visit for acute illness (acute-phase visit), follow-up evaluations occurred at 20 (SD) days (±10 days), 3 months (±10 days), 6 months (±15 days), and 12 months (±30 days). At the acute-phase visit and at all follow-up visits, a blood sample was obtained.

For every participant, we attempted to determine the cause of infection by testing acute-phase serum for virus in *Ae. albopictus* (C6/36) and African green monkey kidney (Vero 76) cell culture (with immunofluorescence assay) and for viral nucleic acid by reverse transcription PCR (RT-PCR). Capture IgM and IgG ELISAs were performed at 1:100 dilution on the acute-phase and all follow-up samples to evaluate antibody responses to MAYV and other endemic arboviruses (i.e., Venezuelan equine encephalitis, Oropouche, group C, Guaroa, and dengue viruses) (*6*). Samples with detectable IgM or IgG were serially diluted and retested. Seroconversion was defined as a >4-fold increase in IgM titer between the acute-phase visit and the second visit. A Mayaro fever case was defined as IgM seroconversion or virus detected by isolation or by RT-PCR. In addition, we collected throat swabs from participants with pharyngeal erythema at the acute-phase visit and urine samples from the 3-month follow-up visit to determine the presence of MAYV with RT-PCR (*13*).

Of 2,094 febrile participants enrolled, 16 (0.8%) had Mayaro fever (Table 1). Of the 16 persons with Mayaro fever, 11 had MAYV isolated by the cell culture assays (11 in both Vero 76 and C6/36), 13 were MAYV positive by RT-PCR, and all had IgM ELISA seroconversion between the acute-phase and 20-day follow-up visits (Table 1). In all 16 participants, no IgM ELISA seroconversion occurred for endemic non-alphavirus viruses (i.e., Oropouche, group C, Guaroa, and dengue viruses). Four participants demonstrated IgM ELISA seroconversion against another alphavirus, Venezuelan equine encephalitis virus, but these 4 all had MAYV identified by immunofluorescence assay and by RT-PCR. Using RT-PCR, we did not detect MAYV in 2 acute visit throat swabs, any second-visit (20-day) serum samples, and any third-visit (3-month) urine samples.

**Table 1:** Demographic factors and laboratory findings at 5 encounters for patients with MAYV infection, Amazon Basin region, Peru, 2010–2013*

Patient	Age, y/sex	Day of illness at enrollment	Isolation	RT-PCR†	IgM; IgG‡				
					Acute phase	Day 20	Month 3	Month 6	Month 12
1	12/F	2	MAYV	MAYV	0; 0	6,400; 100	0; 6,400	0; 25,600	0; 25,600
2	28/F	2	MAYV	MAYV	0; 0	1,600; 100	0; 1,600	0; 6,400	0; 25,600
3	19/F	4	Neg	Neg	0; 0	6,400; 100	0; 1,600	–	–
4	11/M	2	MAYV	MAYV	0; 0	1,600; 100	0; 1,600	0; 1,600	0; 6,400
5	41/F	2	MAYV	MAYV	0; 0	25,600; 400	0; 1,600	0; 1,600	0; 6,400
6	20/M	2	Neg	Neg	0; 0	6,400; 1,600	0; 1,600	0; 400	–
7	36/F	1	MAYV	MAYV	0; 0	6,400; 100	0; 102,400	0; 409,600	0; 102,400
8	35/F	1	MAYV	MAYV	0; 0	1,600; 100	400; 1,600	0; 25,600	0; 25,600
9	43/M	2	MAYV	MAYV	0; 0	1,600; 0	0; 1,600	0; 6,400	0; 6,400
10	34/F	3	MAYV	MAYV	0; 0	6,400; 100	0; 1,600	0; 6,400	0; 25,600
11	46/F	3	Neg	MAYV	0; 0	1,600; 400	0; 6,400	–	–
12	51/M	4	Neg	Neg	0; 0	6,400; 400	0; 1,600	0; 6,400	0; 6,400
13	40/F	1	MAYV	MAYV	0; 0	1,600; 400	400; 25,600	0; 25,600	0; 25,600
14	11/M	2	MAYV	MAYV	0; 0	1,600; 400	0; 25,600	0; 6,400	0; 6,400
15	11/M	3	MAYV	MAYV	0; 0	1,600; 400	0; 6,400	0; 25,600	0; 25,600
16	64/M	2	Neg	MAYV	0; 0	6,400; 25,600	400; 6,400	0; 6,400	0; 6,400

*MAYV, Mayaro virus; RT-PCR, reverse transcription PCR; Neg, negative; –, visits not attended by the patient. †Isolation and RT-PCR results are from the acute-phase visit. ‡For ELISA IgM and IgG results, endpoint titration values were determined. All serology values are expressed as inverse titers.

Besides fever, the most common symptoms affecting participants in the acute stage of MAYV infection were malaise, headache, arthralgia, myalgia, and retro-orbital pain. The prevalence of these and other nonjoint signs and symptoms at the acute-phase and follow-up visits are available in the Technical Appendix Table.

**Figure 2:**
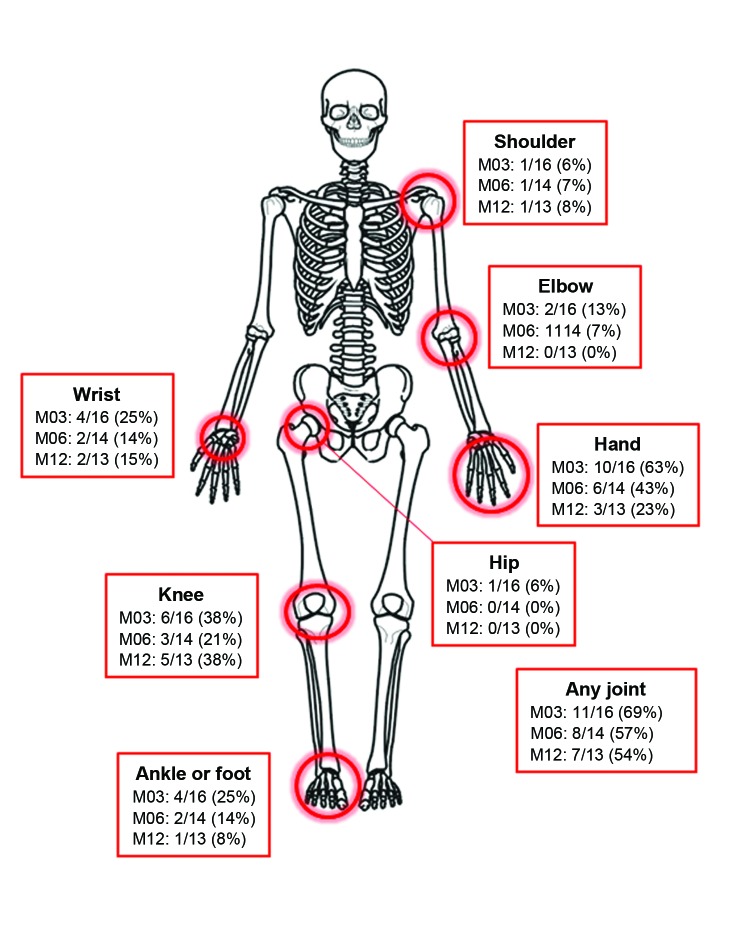
Prevalence of joint involvement at the different follow-up visits for Mayaro virus infection, Amazon Basin region, Peru, 2010–2013. Denominators varied because of varying numbers of participants reporting to each follow-up visit. M03, 3-month follow-up; M06, 6-month follow-up; M12, 12-month follow-up.

Although reports of joint pain waned in study participants by the second (20-day) visit, complaints increased at 3 months and persisted in 54% even after 12 months. Joints of the hand, wrist, elbow, feet, and knee were identified as problematic, whereas hip or axial joint pain was rare (Figure 2). The chronic joint pain often interfered with activities of daily living (Table 2).

**Table 2:** Specific limitations and length of time of limitation caused by long-term joint pain in 16 patients with Mayaro virus infection, Peru, 2010–2013

Patient	Occupation	Limitation secondary to long-term joint pain	Duration of limitation, mo
1	Student	Inability to write because of pain and stiffness in finger joints of both hands; inability to walk long distances because of pain in knees and ankles	12
2	Office worker	No long-term limitations	NA
3	Housewife	No long-term limitations at 3 mo; lost to follow-up at 6 mo	NA
4	Student	No long-term limitations	NA
5	Housewife	No long-term limitations	NA
6	Soldier	No long-term limitations at 6 mo; lost to follow-up at 12 mo	NA
7	Housewife	Limited ability to perform housework because of pain in hand and wrist joints	3
8	Secretary	Inability to remain seated for long periods	8
9	Electrician and other manual labor	Limited ability to climb electric poles because of pain and stiffness in both hands and elbows	12
10	Teacher	Inability to lecture standing up for prolonged periods because of pain in feet and knees	12
11	River boat cook	Limited ability to cook because of pain in both hands, wrists, and knees at 3 mo; lost to follow-up at 6 mo	3
12	Teacher	Difficulty writing because of pain in both hands and arms and pain in shoulders with movement	12
13	Teacher	Inability to lecture standing up for prolonged periods because of pain in hands, knees, and ankles	6
14	Student	Inability to write because of pain and stiffness in finger joints of both hands	6
15	Student	Moderate pain when playing sports, such as basketball	3
16	Driver	No specific limitations noted	NA

*NA, not applicable.

## Conclusions

This study demonstrated that persons with acute Mayaro fever often have many nonspecific symptoms but may continue to have chronic joint pain for at least 1 year after acute illness. Our study offers physicians valuable prognostic data to share with patients. It also indicates the need to consider MAYV infection in patients with seronegative arthritis (i.e., negative rheumatoid factor and antinuclear antibodies) in regions to which MAYV is endemic.

Previous reports have documented persistent joint pain after MAYV infection (*2*–*5*,*9*,*11*), although all of these were either solitary case reports or case series of <4 persons. By using follow-up periods ranging from 1 month to 12 months, most of these studies identified persistent symptoms in the fingers. Involvement of joints of the wrist, ankle, and knee also were mentioned, similar to the participants in our study.

Long-term manifestations of infection with other alphaviruses have been more robustly characterized, with persistent arthralgia being commonly described. Follow-up of chikungunya virus–infected patients on Réunion Island revealed that ≈60% had joint pain >3 years after acute illness that most often affected the fingers, wrists, knees, and ankles (*14*). Sindbis virus infection in a cohort in Finland resulted in persistent arthralgia lasting at least a year in half of those infected, with ankles, fingers, and wrists being most often affected (*15*). One caveat of our study and other studies is the difficulty in definitively attributing persistent arthralgia solely to viral infection, although our participants’ limitations in activities of daily living were all described as starting after their acute Mayaro fever illness.

IgM seroconversion occurred in all of the participants in our study that were identified with either isolation or RT-PCR, consistent with another report that noted the reliability of serology in detecting MAYV infection (*9*). In our study participants identified by serology, both RT-PCR and culture were more sensitive than what others have found for Sindbis virus infection, for which 1 study found sensitivities of 7% and 1%, respectively (*15*). However, RT-PCR and culture were negative in the only 2 participants in our study who had Mayaro fever after day 3 of symptoms, suggesting a narrow window when these assays may be effective.

No effective vaccine or antiviral agent exists for the arthritogenic alphaviruses, and treatment relies mainly on supportive modalities, such as nonsteroidal anti-inflammatory medications (*12*). Our results offer evidence that MAYV, similar to other alphaviruses, may cause protracted joint symptoms and provide further impetus to the development of more effective preventive and treatment strategies.

Technical Appendix Signs and symptoms in patients with Mayaro virus infection at the acute-phase visit and each follow-up visit, Amazon Basin region, Peru, 2010–2013.

## References

[1] Anderson CR, Downs WG, Wattley GH, Ahin NW, Reese AA. Mayaro virus: a new human disease agent. II. Isolation from blood of patients in Trinidad, B.W.I. Am J Trop Med Hyg. 1957;6:1012–6 . 13487973 10.4269/ajtmh.1957.6.1012

[2] Talarmin A, Chandler LJ, Kazanji M, de Thoisy B, Debon P, Lelarge J, Mayaro virus fever in French Guiana: isolation, identification, and seroprevalence. Am J Trop Med Hyg. 1998;59:452–6 . 9749643 10.4269/ajtmh.1998.59.452

[3] Karbaat J, Jonkers AH, Spence L. Arbovirus infections in Dutch military personnel stationed in Surinam: a preliminary study. Trop Geogr Med. 1964;16:370–6 . 14265514

[4] Torres JR, Russell KL, Vasquez C, Barrera R, Tesh RB, Salas R, Family cluster of Mayaro fever, Venezuela. Emerg Infect Dis. 2004;10:1304–6 . 10.3201/eid1007.030860 15324555 PMC3098555

[5] Tesh RB, Watts DM, Russell KL, Damodaran C, Calampa C, Cabezas C, Mayaro virus disease: an emerging mosquito-borne zoonosis in tropical South America. Clin Infect Dis. 1999;28:67–73 . 10.1086/515070 10028074

[6] Forshey BM, Guevara C, Laguna-Torres VA, Cespedes M, Vargas J, Gianella A, Arboviral etiologies of acute febrile illnesses in western South America, 2000–2007. PLoS Negl Trop Dis. 2010;4:e787. 10.1371/journal.pntd.0000787 20706628 PMC2919378

[7] Mourão MP, Bastos Mde S, de Figueiredo RP, Gimaque JB, Galusso Edos S, Kramer VM, Mayaro fever in the city of Manaus, Brazil, 2007–2008. Vector Borne Zoonotic Dis. 2012;12:42–6 . 21923266 10.1089/vbz.2011.0669 PMC3249893

[8] Azevedo RS, Silva EV, Carvalho VL, Rodrigues SG, Nunes-Neto JP, Monteiro H, Mayaro fever virus, Brazilian Amazon. Emerg Infect Dis. 2009;15:1830–2. 10.3201/eid1511.090461 19891877 PMC2857233

[9] Pinheiro FP, Freitas RB, Travassos da Rosa JF, Gabbay YB, Mello WA, LeDuc JW. An outbreak of Mayaro virus disease in Belterra, Brazil. I. Clinical and virological findings. Am J Trop Med Hyg. 1981;30:674–81 . 6266263 10.4269/ajtmh.1981.30.674

[10] Long KC, Ziegler SA, Thangamani S, Hausser NL, Kochel TJ, Higgs S, Experimental transmission of Mayaro virus by *Aedes aegypti.* Am J Trop Med Hyg. 2011;85:750–7 . 10.4269/ajtmh.2011.11-0359 21976583 PMC3183788

[11] Hassing RJ, Leparc-Goffart I, Blank SN, Thevarayan S, Tolou H, van Doornum G, Imported Mayaro virus infection in the Netherlands. J Infect. 2010;61:343–5. 10.1016/j.jinf.2010.06.009 20600300

[12] Suhrbier A, Jaffar-Bandjee MC, Gasque P. Arthritogenic alphaviruses—an overview. Nat Rev Rheumatol. 2012;8:420–9. 10.1038/nrrheum.2012.64 22565316

[13] Powers AM, Aguilar PV, Chandler LJ, Brault AC, Meakins TA, Watts D, Genetic relationships among Mayaro and Una viruses suggest distinct patterns of transmission. Am J Trop Med Hyg. 2006;75:461–9 . 16968922

[14] Schilte C, Staikovsky F, Couderc T, Madec Y, Carpentier F, Kassab S, Chikungunya virus–associated long-term arthralgia: a 36-month prospective longitudinal study. PLoS Negl Trop Dis. 2013;7:e2137. 10.1371/journal.pntd.0002137 23556021 PMC3605278

[15] Kurkela S, Manni T, Myllynen J, Vaheri A, Vapalahti O. Clinical and laboratory manifestations of Sindbis virus infection: prospective study, Finland, 2002–2003. J Infect Dis. 2005;191:1820–9. 10.1086/430007 15871114

