# Supplementary material for: Mayaro Virus Infection, Amazon Basin Region, Peru, 2010–2013
Source: Emerg Infect Dis. 2013 Nov;19(11):1839–42. doi: 10.3201/eid1911.130777 (PMC3837653; doi:10.3201/eid1911.130777)
Supplement: Technical Appendix — Signs and symptoms in patients with Mayaro virus infection at the acute-phase visit and each follow-up visit, Amazon Basin region, Peru, 2010–2013. [file 13-0777-Techapp-s1.pdf]

Article DOI: http://dx.doi.org/10.3201/eid1911.130777

Mayaro Virus Infection, Amazon Basin Region, Peru, 2010–2013

Technical Appendix

Technical Appendix Table. Signs and symptoms in 16 patients with Mayaro virus infection at the acute-phase and each follow-up visit, Amazon Basin region, Peru, 2010–2013.

Sign or symptom	Acute-phase visit	Day 20	Month 3	Month 6	Month 12
Malaise	16/16	2/16	4/16	1/14	2/13
Arthralgia	15/16	3/16	11/16	8/14	7/13
Headache	15/16	2/16	6/16	4/14	4/13
Myalgia	14/16	1/16	1/16	2/14	2/13
Pain behind eyes	12/16	0/16	1/16	1/14	0/13
Anorexia	12/16	0/15	1/16	0/14	1/13
Nausea	11/16	0/15	1/16	0/14	0/13
Vomiting	11/16	0/15	1/16	0/14	0/13
Dizziness	10/16	1/16	1/16	1/14	2/13
Dysgeusia	10/16	0/15	0/16	0/14	1/13
Abdominal pain	8/16	1/15	1/16	0/14	0/13
Rash	8/16	0/15	0/16	0/14	0/13
Sore throat	4/16	0/16	1/16	1/14	0/13
Photophobia	4/15	0/16	1/16	0/14	0/13
